# Molecular Resources from Transcriptomes in the Brassicaceae Family

**DOI:** 10.3389/fpls.2017.01488

**Published:** 2017-08-29

**Authors:** Lua Lopez, Eva M. Wolf, J. Chris Pires, Patrick P. Edger, Marcus A. Koch

**Affiliations:** ^1^Biodiversity and Plant Systematics, Centre of Organismal Studies, University of Heidelberg Heidelberg, Germany; ^2^Division of Biological Sciences, University of Missouri Columbia, MO, United States; ^3^Department of Horticulture, Michigan State University East Lansing, MI, United States; ^4^Ecology, Evolutionary Biology and Behavior, Michigan State University East Lansing, MI, United States

**Keywords:** Brassicaceae, RNAseq/transcriptome, SSRs, molecular resources, data mining, sequencing platform

## Abstract

The rapidly falling costs and the increasing availability of large DNA sequence data sets facilitate the fast and affordable mining of large molecular markers data sets for comprehensive evolutionary studies. The Brassicaceae (mustards) are an important species-rich family in the plant kingdom with taxa distributed worldwide and a complex evolutionary history. We performed Simple Sequence Repeats (SSRs) mining using *de novo* assembled transcriptomes from 19 species across the Brassicaceae in order to study SSR evolution and provide comprehensive sets of molecular markers for genetic studies within the family. Moreover, we selected the genus *Cochlearia* to test the transferability and polymorphism of these markers among species. Additionally, we annotated *Cochlearia pyrenaica* transcriptome in order to identify the position of each of the mined SSRs. While we introduce a new set of tools that will further enable evolutionary studies across the Brassicaceae, we also discuss some broader aspects of SSR evolution. Overall, we developed 2012 ready-to-use SSR markers with their respective primers in 19 Brassicaceae species and a high quality annotated transcriptome for *C. pyrenaica*. As indicated by our transferability test with the genus *Cochlearia* these SSRs are transferable to species within the genus increasing exponentially the number of targeted species. Also, our polymorphism results showed substantial levels of variability for these markers. Finally, despite its complex evolutionary history, SSR evolution across the Brassicaceae family is highly conserved and we found no deviation from patterns reported in other Angiosperms.

## Introduction

The field of evolutionary biology has never seen such an advance as the one promoted by the recent advent and standardization of next-generation sequencing (NGS) techniques. Now, many unsolved questions can be addressed (Nadeau and Jiggins, 2010). Despite that NGS technologies have drastically decreased their costs since their start, it stills remains cost prohibitive for many research programs. Fortunately, as NGS generates enormous amounts of data its analysis can be approached from multiple perspectives targeting different objectives. It is in this context where sharing data proves to be enormously beneficial to the scientific community. Open resources as those provided by the National Center for Biotechnology Information (NCBI Resource Coordinators, 2016), UniProt (The UniProt Consortium, 2007), or TAIR (Huala et al., 2001) are incredibly valuable to the broader research community. Likewise, countless initiatives and researchers are willing to release their data upon request. The sharing of research data yields multiple benefits; it promotes multiple perspectives, helps identify errors, discourages fraud and increases the efficient use of funding resources by avoiding duplicate data collection (Piwowar et al., 2007).

A popular use of NGS data is the discovery of large numbers of molecular markers for population genetic studies (Pashley, 2006; Wöhrmann et al., 2012; Lopez et al., 2015; Tanwar et al., 2017). The accurate assessment of genetic variation within and across species is pivotal in studies addressing topics like evolution, phylogeny and/or conservation and the larger the marker data set used, the more accurate the results and extrapolations are. Among the plethora of available molecular markers for genetic studies Simple Sequence Repeats (SSR) are extremely popular for several reasons. SSRs are known to display high levels of polymorphism, have a multiallelic behavior, are codominant and abundant along the genome (Morgante and Olivieri, 1992; Ritland, 2000); but, their development is still time consuming and relatively expensive (Squirrell et al., 2003). The alternative use of large genomic and/or transcriptomic datasets speeds and cheapens their design process (Lopez et al., 2015). Moreover, classical SSRs are mainly species-specific and markers developed for a taxon are unlikely exchangeable to another (Barbara et al., 2007) while SSRs from the transcribed portion of the genome are highly transferable between related species, and often even genera (Varshney et al., 2005b; Pashley, 2006; Huang et al., 2014) conferring further advantages in comparisons across related taxa. Ultimately, these SSRs can be considered “functional” markers as they represent a portion of the genome expressed under certain circumstances and a putative function can be associated to the sequences containing SSRs by homology search (Andersen and Lübberstedt, 2003). However, this type of marker may also have potential drawbacks. SSRs developed from the transcribed fraction of the genome might display lower levels of variability compared with classical SSRs (Ellis and Burke, 2007) and they might deviate from neutrality impacting the population structure inferences (Varshney et al., 2005a). However, recent studies pointed out that these markers have levels of polymorphism similar to their classical counterparts and, because only a small fraction of the genome might be subjected to recent positive selection, population structure measurements are comparable to those derived from anonymous SSRs (Tiffin and Hahn, 2002; Woodhead et al., 2005). To date, SSRs derived from the transcribed region of the genome have proved their usefulness in phylogenetic studies (Tabbasam et al., 2013), in comparative genetic mapping studies between species (Yu et al., 2004) and in studies assessing levels of genetic diversity (Wöhrmann and Weising, 2011; Olango et al., 2015).

Understanding how evolutionary forces shaped the current patterns of biodiversity worldwide is a challenging but exciting research focus. In the plant kingdom, the Brassicaceae are a fascinating model system for evolutionary studies for many reasons. First, it is one of the most diverse and geographically widespread plant families (Koch and Kiefer, 2006; Lysak and Koch, 2011; Al-Shehbaz, 2012). It compromises ~3,990 species in 52 tribes and more than 325 genera mostly distributed in temperate regions of the world, with scarce representatives in subtropical regions (Koch et al., 2012; Kiefer et al., 2014). Second, it includes many key species because of their economical and/or research value. The first plant with its whole genome sequenced, *Arabidopsis thaliana*, is a well-known representative of the Brassicaceae and numerous advances of the current knowledge of plant biology have been made from its studies (Meinke et al., 1998; Hohmann et al., 2014; Novikova et al., 2016). Likewise, this family includes many important agricultural and horticultural members like species from the genera *Brassica, Camelina*, and *Raphanus*. Third, the Brassicaceae net diversification rates are among the highest inferred for terrestrial plants (Jordon-Thaden et al., 2013; Karl and Koch, 2013; Hohmann et al., 2015). Moreover, recurrent polyploidization has played a significant role in the Brassicaceae evolution with circa half of its taxa hypothesized to have a recent polyploid origin (Franzke et al., 2011; Lysak and Koch, 2011; Hohmann et al., 2015) and the entire family having undergone a unique polyploid event not shared with other Brassicales families (Barker et al., 2009; Edger et al., 2015). Finally, initiatives such as, the BrassiBase knowledge database facilitates and promotes studies targeting the Brassicaceae family by providing publicly available resources (Kiefer et al., 2014). Despite these major advances and resources, multiple aspects of the evolutionary trajectory and phylogeny of this family still remain controversial or unresolved. In this regard, the lack of a sizable data-set of transferable and reliable molecular markers to accurately infer levels of genetic variation and divergence across species has limited the use of the Brassicaceae as an evolutionary model family.

The present study aims to fill this gap by developing a vast number of SSRs with their primers from RNAseq data of 19 Brassicaceae species covering all major evolutionary lineages within the family and an outgroup species from the sister family (*Cleome violacea*; Cleomaceae). These markers are publicly available in the BrassiBase portal (Kiefer et al., 2014). Our RNAseq dataset results from the fruitful collaboration and sharing of scientific data between researchers and initiatives; data originally used in (Huang et al., 2016) and data provided by the 1,000 plants initiative (1KP; Johnson et al., 2012). RNAseq data of each species was *de novo* assembled and the resulting transcriptomes went through rigorous quality checks before being mined for SSRs. We tested SSRs polymorphism and transferability *in silico* across eight *Cochlearia* species. Also, we annotated the *de novo* assembled transcriptome for *Cochlearia* so the sequences containing SSRs could be identified as coding or non-coding and a putative function could be assigned to those SSRs located in coding regions. Finally, we checked for evolutionary patterns of SSR types and motifs across the entire Brassicaceae phylogeny.

## Materials and methods

### Sequence data sources

**Figure 1:**
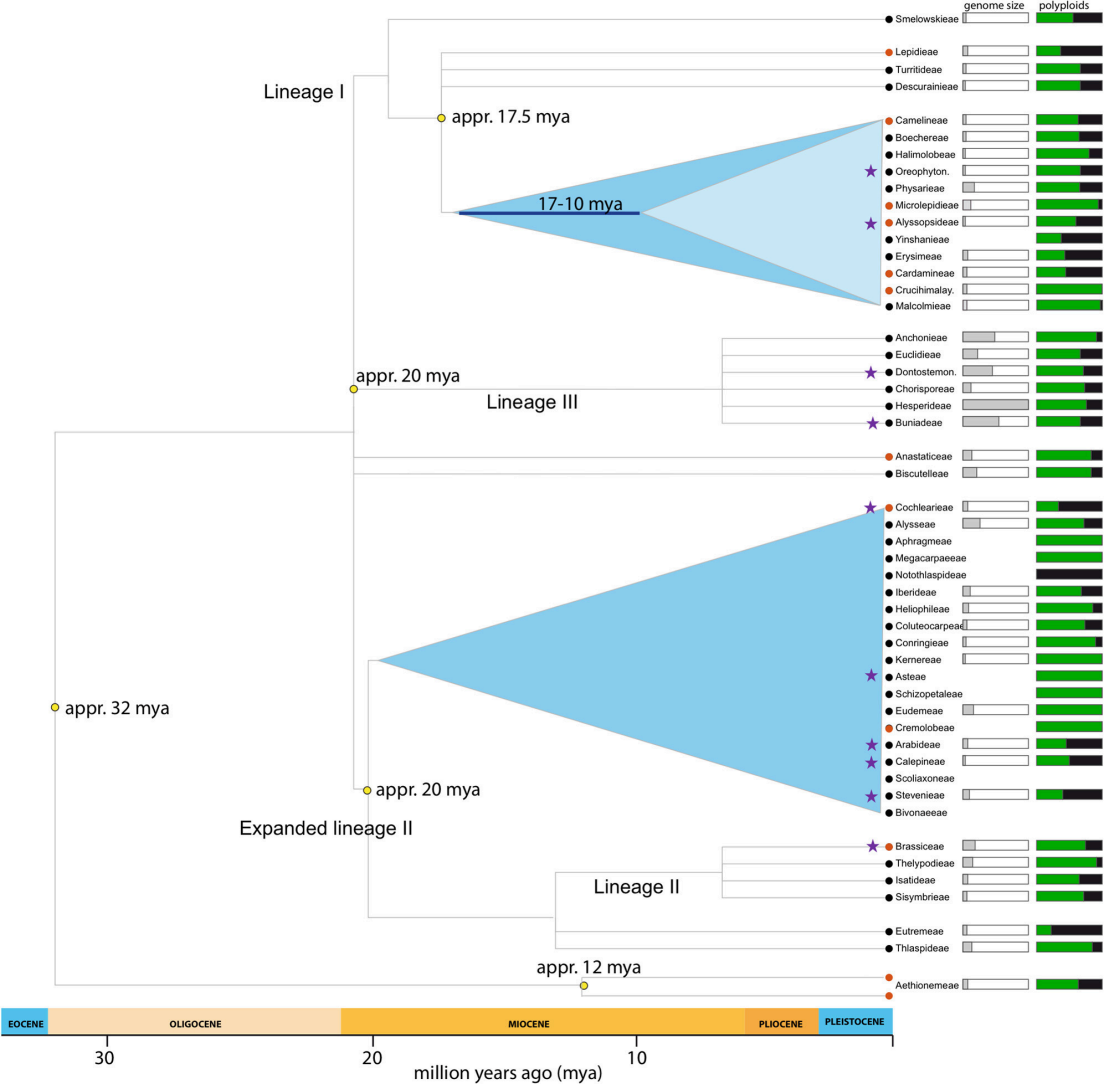
Brassicaceae lineages included in the present study. Phylogeny adapted from Franzke et al. (2011). Purple stars marks tribes with samples included in this study. Genome size (1 Cx-value) indicates mean tribal value (whole bar = 4.33pg). Percentage of polyploids indicates neopolyploids (auto- and allopolyploids; Hohmann et al., 2014).

This study uses a set of 19 species (Table 1) covering all major lineages of the Brassicaceae phylogeny (Figure 1). Our data set was kindly provided through two collaborations. Ten out of the nineteen species RNAseq data were provided by Huang et al. (2016). For these RNA extraction, library preparation and sequencing were performed as specified in Huang et al. (2016). The renaming nine species were facilitated by the 1KP initiative. Protocol for RNA extraction, library preparation and sequencing details are described in Johnson et al. (2012).

**Table 1:** Species' information table.

**Species**	**Tribe**	**Lineage**	**Source**	**Voucher**	**SP**
*Murbeckiella boryi* (Boiss.) Rothm.	Oreophytoneae	I	1	none	HiSeq2000
*Alyssopsis mollis* (Jacq.) O.E.Schulz	Alyssopsideae	I	1	none	HiSeq2000
*Calepina irregularis* (Asso) Thell.	Calepineae	Exp-II	1	none	HiSeq2000
*Cochlearia pyrenaica* DC.	Cochlearieae	Exp-II	1	none	HiSeq2000
*Kernera saxatilis* (L.) Sweet	Kernereae	Exp-II	1	none	HiSeq2000
*Bunias orientalis* L.	Buniadeae	III	1	none	HiSeq2000
*Clausia aprica* (Stephan ex Willd.)Trotzky	Dontostemoneae	III	1	none	HiSeq2000
*Macropodium nivale* R.Br.	Stevenieae	Exp-II	1	none	HiSeq2000
*Microthlaspi perfoliatum* (L.) F.K.Mey	Coluteocarpeae	Exp-II	1	none	HiSeq2000
*Noccaea caerulescens* (J. Presl & C. Presl) F.K.Mey	Coluteocarpeae	Exp-II	1	none	HiSeq2000
*Arabis alpina* L.	Arabideae	Exp-II	2 (TZWR)	PPE-2012-1000-0003 (UMO)	HiSeq
*Brassica nigra* W.D.J.(Koch)	Brassiceae	II	2 (IPWB)	PPE-2012-1000-0001 (UMO)	HiSeq
*Cleome violacea* (L.) Raf.	Cleomaceae	outgroup	2 (HELY)	PPE-2012-1000-0004 (UMO)	HiSeq
*Sinapis alba* L.	Brassiceae	II	2 (VMNH)	none	HiSeq
*Draba aizoides* L.	Arabideae	Exp-II	2 (HABV)	none	HiSeq
*Draba hispida* Willd.	Arabideae	Exp-II	2 (GTSV)	none	HiSeq
*Draba magellanica* Lam.	Arabideae	Exp-II	2 (UVQL)	none	HiSeq
*Draba ossetica* (Rupr.) Sommier & Levier	Arabideae	Exp-II	2 (LIQF)	none	HiSeq
*Draba sachalinensis* (Schmidt) Trautv.	Arabideae	Exp-II	2 (BXBF)	none	HiSeq

*Tribe, tribe which the species belongs to; Lineage, phylogenetic lineage to which the species belong within the Brassicaceae phylogeny as in Hohmann et al. (2015) note that Exp-II means expanded lineage II; source, 1 indicates sequences from Huang et al. (2016) and 2 from the 1 KP (1 KP sample ID); voucher, ID of those specimens that are registered in an herbarium*.

### Data preprocessing and *de novo* assembly

Prior to assembly, we filtered and trimmed the raw paired-end reads using Trimmomatic v0.36 (Bolger et al., 2014) based on the following criteria. We checked for remaining adapter sequences in the reads. In Trimmomatic we looked for seed matches allowing a maximum of two mismatches and these seeds were extended and clipped when paired end reads had a score of 30. Moreover, we removed leading and trailing bases of low quality (<20) or Ns. Likewise, we cut and removed the 3′ end if the quality of a 4-base wide sliding window dropped below 15. Finally, we discarded reads that after these steps were shorter that 50 bases long. By implementing this stringent filtering process, we ensured that only high-quality paired-end clean reads were used for the assembly. Finally, we further inspected the clean reads using FastQC v 0.11.5 (http://www.bioinformatics.babraham.ac.uk/projects/) to confirm that they met our quality standards.

To recover full-length transcripts we fed the clean reads to the software Trinity for their *de novo* assembly (Grabherr et al., 2011). In this regard, Trinity is and efficient and robust method for the *de novo* reconstruction of transcriptomes from short read sequences using the de Bruijn graph algorithm (Zhao et al., 2011; Honaas et al., 2016; Rana et al., 2016). We conducted all *de novo* assemblies using the default parameters and we assessed the quality of the *de novo* assembled transcriptome as it has a large impact on the accuracy of the subsequent analyses. First, we examined the read representation of the assembly by mapping the raw paired-end reads back to the assembly contigs using the bowtie aligner (Langmead et al., 2009). Second, we evaluated the completeness of the assembly in terms of conserved ortholog content with BUSCO (Simão et al., 2015).

### SSR mining and primer design

For SSRs detection we used QDD v.3.1.2 (Meglecz et al., 2010; Meglécz et al., 2014). We inputted the assembled contigs to QDD3 and screened them for SSRs. Our search was restricted to perfect SSRs with a minimum length of 20 bases. Which, in terms of repeats translates into 10 repeats for di-, seven for tri-, five for tetra- and four for penta- and hexa-nucleotides, respectively. We excluded mononucleotides from the mining criteria because interpreting their polymorphism is challenging. Besides, in order to have enough flanking sequence for primer design, during SSR searches we only took into account contigs larger than 100 bases. For primers' design we used the version of Primer3 embedded in QDD1 (Rozen and Skaletsky, 2000). Our primer parameters were as follows: length of primers ranging from 18 to 23 nucleotides (optimum 20 bases), annealing temperature 55–65°C (optimum 60°C), GC content 30–70% (optimum 50%), and PCR product size from 90 to 320 bases. Also, during the primer search we specified that no other target SSR was allowed in the flanking region. Finally, we only selected those SSRs where their PCR product presented no overlapping with other SSRs sequences.

### Compositional analysis of SSR mining

Because the data was sequenced on two different versions of the Illumina HiSeq we ran an exploratory analysis aiming to identify the impact that the sequencing platform might have over the results output. We evaluated the role of data source in raw reads, number of assembled contigs and number of mined SSRs. Once we identified the influence of the data source we proceeded to investigate the occurrence and frequency of SSR motifs. For this purpose, we imported the QDD output files into RStudio and using a combination of sorting and counting functions we summarized: repeat types, number of repeats, and frequency for each species. Besides, we also sorted out these categories based on phylogenetic lineages trying to look for evolutionary patterns. We chose tabular and graphical representations for results display.

### Transcriptome annotation of *Cochlearia pyrenaica*

Using Blast2GO (Conesa et al., 2005), a comprehensive software designed for the functional annotation and analysis of gene and protein sequences, we annotated the assembled contigs. We compared all transcripts with various databases aiming to extract the maximum possible information based on sequence and functional similarity. We used the BLASTX algorithm to search for homologous sequences (e-value cut off 1.0E-5) against the UniProtKB (both SwissProt and TrEMBL) and NCBI non-redundant databases and we functionally annotated the transcripts according to the Gene Ontology nomenclature (Gene Ontology, the Gene Ontology Consortium, 2000; http://www.geneontology.org). Moreover, we identified conserved motifs/domains through InterProScan. In order to obtain a comprehensive integrated annotation result, we also retrieved GO terms from the InterProScan ID's and merged them with our blast derived GO annotations. To maximize the information included on the final transcriptome annotation we also conducted a search in the Rfam database and performed an enzyme code and KEGG pathway annotation. First, the Rfam database contains information of non-coding RNAs (Griffiths-Jones, 2003; Nawrocki et al., 2015) and incorporating this information provides a more comprehensive annotation. Second, the Kyoto Encyclopedia of Genes and Genomes (KEGG) database is used extensively to reveal molecular interaction network and metabolic pathways (Kanehisa and Goto, 1999). In the last step, we performed a GO_Slim reduction on GO terms aiming to obtain more precise GO definitions. In all the steps involving Blast2GO we kept the default parameters: InterProScan analysis, Rfam search, enzyme code/KEGG annotation, and GO_Slim. Finally, we identified putative coding regions (Open Reading Frame) in the transcripts using Transdecoder (http://transdecoder.sf.net). The latter uses the information of the BLASTX in order to obtain a more accurate prediction of the ORFs.

### SSRs transferability and polymorphism in the genus *Cochlearia*

We crossed the SSRs mined from the *Cochlearia pyrenaica* transcriptome with its annotation to identify their putative position within the transcriptome (i.e., coding and non-coding regions). We stablished that a SSR was within coding region when all its bases were within an ORF. Likewise, we considered a SSR as non-coding when it was fully positioned outside the ORF. To perform the *in silico* transferability and polymorphism analysis we selected 15 SSR which we tested in a total of 14 individuals encompassing eight *Cochlearia* species (Table 2). We mapped previously filtered high quality genomic reads to the transcripts containing SSRs. For the mapping we used the BWA-MEM algorithm in in BWA v0.7.5a (Li and Durbin, 2010) with default parameters except for the unpaired read pair penalty which was set to 15. Moreover, we removed ambiguously mapped and duplicate reads using samtools v.0.1.18 (Li et al., 2009; Li, 2011) and performed a local realignment around indels with the GATK (McKenna et al., 2010) “IndelRealigner” tool with default settings. We loaded the mappings in Geneious R9 (http://www.geneious.com; Kearse et al., 2012) where the SSRs polymorphism was manually assessed at intra- and inter-species level. Finally, we computed within and across species diversity indexes, as well as a Principal Component Analysis (PCoA) based on standardized genetic distanced between individuals with GenAlex v6.503 (Peakall and Smouse, 2006, 2012).

**Table 2:** Species from the genus *Cochlearia* used for the polymorphism study.

**ID**	**Voucher**	**Species**	**Location**
Caes0741	RBGE Gill Stelle s.n.	*C. aestuaria* (Lloyd) Heywood	Asturias, Spain.
Calp0759	HEID BNr. 504202	*C. alpina* (Bab.) H.C.Watson	Teesdale, UK.
Cexc1253	HEID BNr. 404206	*C. excelsa* Zahlbr. Ex Fritsch	Mt. Seckauer Zinken, Austria.
Cgro0474	O 894768	*C. groenlandica* L.	Svalbard, Norway.
Cgro1038	O 990848	*C. groenlandica* L.	Nome Census Area, Alaska.
Cisla1233	HEID BNr.503296	*C. islandica* Pobed	Stokkseyri, Iceland.
Cpyr0260	HEID BNr.501471	*C. pyrenaica* DC.	Kelmis, Belgium.
Cpyr0310	HEID BNr.480392	*C. pyrenaica* DC.	Türnitz, Austria.
Cpyr0456	HEID BNr.503877	*C. pyrenaica* DC.	Carpathian Mountains, Slovakia.
Cpyr0560	HEID BNr.921491	*C. pyrenaica* DC.	Allgäu, Germany.
Cpyr0699	HEID RBGE Gill Stelle GS8	*C. pyrenaica* DC.	Gordale Scar, Scotland.
Cpyr1222	HEID BNr.503293	*C. pyrenaica* DC.	Asturias, Spain.
Cses1285	HEID BNr.503294	*C. sessilifolia* Rollins	Kodiak Island, Alaska.
Ctri1287	MT MT00127822	*C. tridactylites* Banks ex DC.	L'Anse Amour, Canada.

*ID, individual ID used to identify each sample in this study; Voucher, herbarium acronym and number identifying the herbarium voucher, Species, species name and Location, name of the location and country where the individual was collected*.

## Results

### Data preprocessing and *de novo* assembly

In this study, we used 19 species' libraries sequenced with Illumina technology. All libraries had the same sequencing depth except for *Cochelaria pyrenaica*, which was sequenced using two lanes in order to obtain higher coverage of the transcriptome for its annotation. Therefore, the number of reads for *C. pyrenaica* greatly exceeded those of the other libraries (110,411,546 total reads). For the remaining 18 species, the number of raw reads differed across libraries, ranging from 9,196,502 reads to 26,930,913 paired-reads for *C. violacea* and *Clausia aprica*, respectively (Table 3). We obtained high quality clean reads after applying a conservative trimming filter which removed adapter sequences, short reads and low quality reads from raw sequence data. The number of high quality clean sequence varied with a minimum of 8,915,641 reads to a maximum of 25,034,919 reads belonging to *Draba ossetica* and *C. aprica*, respectively (Table 3). We carried out the subsequent *de novo* assembly for each data using the clean reads. As expected, the number of assembled transcripts differed across species. The assembly of *Microthlaspi perfoliatum* rendered the smallest number of transcripts (35,086) while *Macropodium nivale* produced the largest assembly in terms of total contigs (97,523). Even if the starting number of clean reads for the *de novo* assembly was much higher in *C. pyrenaica*, the number of transcripts that we obtained was not the highest suggesting that other factors, besides number of input reads, might play a role during the assembly including RNA quality (Johnson et al., 2012).

**Table 3:** Results' summary for each species.

**Species**	**1C**	**SP**	**Reads**	**Trimmed**	**Contigs**	**Bowtie**	**BUSCO**	**SSR**	**Density**	**SSR+P**.
*Murbeckiella boryi* (Boiss.) Rothm.	0.2	HiSeq2000	14771107	13824138	40433	87.55	92.26	1005	1/21.99	128
*Alyssopsis mollis* (Jacq.) O.E.Schulz	0.19	HiSeq2000	19418002	18035058	44010	82.25	94.25	1808	1/13.51	111
*Calepina irregularis* (Asso) Thell.	0.25	HiSeq2000	16020733	14949117	51415	73.73	93.62	1516	1/16.08	100
*Cochlearia pyrenaica* DC.	0.4	HiSeq2000	110411546	102682738	51572	83.91	94.46	2783	1/12.25	160
*Kernera saxatilis* (L.) Sweet	0.2	HiSeq2000	14995455	13932451	41932	86.53	92.78	1316	1/35.28	115
*Bunias orientalis* L.	2.63	HiSeq2000	20013688	18704854	60941	82.92	94.67	1533	1/20.71	112
*Clausia aprica* (Stephan ex Willd.) Trotzky	3.97	HiSeq2000	26930913	25034919	71021	77.97	94.25	1418	1/23.51	96
*Macropodium nivale* R.Br.	0.53	HiSeq2000	23012277	21347707	97523	72.75	95.19	2524	1/15.22	128
*Microthlaspi perfoliatum* (L.) F.K.Mey	0.26	HiSeq2000	16766557	15823266	35086	90.13	89.44	1294	1/15.01	135
*Noccaea caerulescens* (J. Presl & C. Presl) F.K.Mey	0.33	HiSeq2000	11364197	11364197	49653	86.40	92.47	2095	1/11.77	130
*Arabis alpina* L.	0.38	HiSeq	10881467	10713549	44322	90.27	90.27	795	1/19.87	110
*Brassica nigra* W.D.J. (Koch)	0.64	HiSeq	12275029	12118888	56228	**52.56**	80.33	691	1/20.58	130
*Cleome violacea* (L.) Raf.	NA	HiSeq	9196502	9048135	40529	82.42	89.33	1448	1/11.67	97
*Draba aizoides* L.	NA	HiSeq	9645203	9335024	40118	80.73	61.19	415	1/18.80	81
*Draba hispida* Willd.	NA	HiSeq	10148457	9768317	36938	82.19	62.45	418	1/18.97	72
*Draba magellanica* Lam.	0.66	HiSeq	11335649	11131989	53209	**57.89**	58.16	434	1/22.11	64
*Draba ossetica* (Rupr.) Sommier & Levier	NA	HiSeq	9270733	8915641	39175	77.89	62.76	448	1/18.69	61
*Draba sachalinensis* (Schmidt) Trautv.	0.42	HiSeq	15233675	14973184	63087	**58.28**	78.77	717	1/20.89	80
*Sinapis alba* L.	0.53	HiSeq	16401105	16186125	60288	**55.12**	84.00	768	1/21.51	102

*Seq., type of Illumina sequencing technology used; 1C, holoploid genome size in pg (http://brassibase.cos.uni-heidelberg.de); SP, type of Illumina sequencing platform used; reads, number of pairs of raw reads in each species dataset; trimmed, number of pairs of clean reads after quality trimming; contigs, number of assembled transcripts from trimmed reads; Bowtie, percentage of raw reads properly mapped to the assembled contigs (in bold those with less than 70% of reads mapped properly); BUSCO, measure in percentage of the completeness of the assembly in terms of conserved ortholog content; SSR, total number of SSR found in each species; density, indicated as number of SSR per kb and SSR+P., number of SSR with primers obtained for each species*.

Before SSR search we examined the quality of the *de novo* assembled transcriptomes using two parameters: the representation of reads and the content of conserved orthologs. In a high-quality transcriptome, the majority of its clean reads are mapped back as proper pairs (~70–80%). Circa 80% of our transcriptomes met this requirement and only four species did not (*Brassica nigra, Draba magellanica, Draba chilensis*, and *Sinapis alba*; Table 3). Transcriptomes with highly repetitive content can lead to lower percentage of read pairs mapping uniquely. Thus, in order to determine if we had a low quality or highly repetitive transcriptome, we checked the level of sequence duplication in our samples. When compared with the others, we identified an excess of repetitive content in the clean reads in the four species with low proportions of properly mapped reads. We also noted that these species were sequenced with the old Illumina HiSeq platform and had overrepresented sequences. Moreover, this overrepresentation of sequences was extended to all samples sequenced with the old Illumina platform but none of these overrepresented sequences had a hit with adapters or other sequences involved in the library preparation and/or sequencing processes. When we considered the second quality measurement results, for two of those four species, *B. nigra and S. alba*, we found a large percentage or orthologs present in their transcriptomes further supporting the repetitive content hypothesis. For the other two, *D. magellanica* and *D. chilensis*, the percentage of orthologs we retrieved was lower, around 60%, indicating that these *de novo* assembled transcriptomes were incomplete. Likewise, two other species, *Draba hispida* and *D. magellanica* reported similar low levels of orthologs content. Despite these cases, the overall quality of our *de novo* assembled transcriptomes was very good. Our quality assessment points toward a bias in relevant aspects for SSRs discovery using RNAseq associated with the choice of sequencing platform.

### Compositional analysis of SSR mining in the brassicaceae family

**Figure 2:**
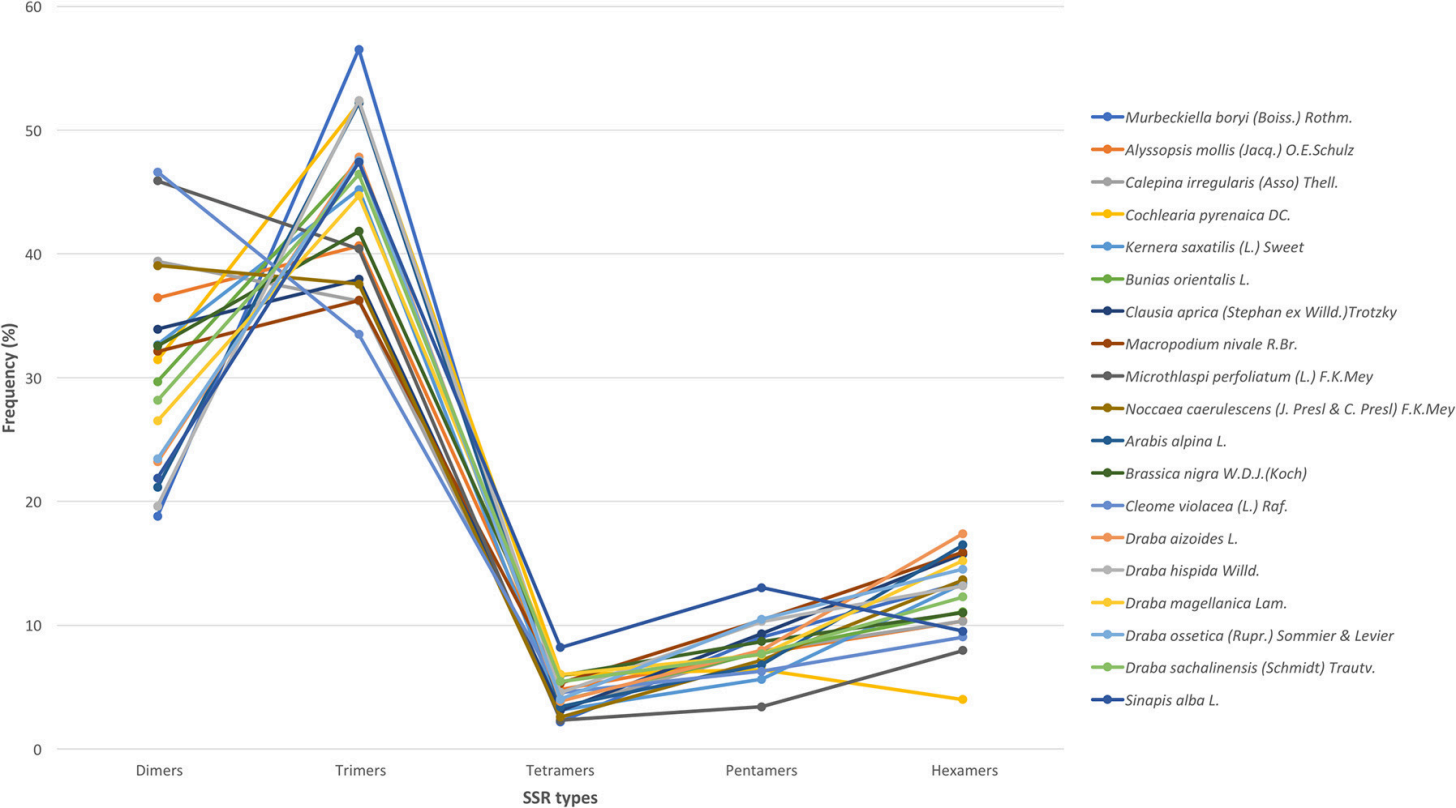
SSR's types distribution across species. In the x-axis, each type of motif is indicated while the y-axis refers to the percentage of each type of motif in each species. Each species is denoted by a color as shown in the legend.

We used all assembled transcripts as input for the SSR search. In total, we found 23,425 SSRs ranging from 2,524 SSRs in *M. nivale* to 415 in *Draba aizoides* (Table 3). The density of SSRs across the *de novo* assembled transcriptomes showed large variation across species. We found that SSRs were distributed more densely in *Noccaea caerulescens* and *C. violaceae* with 1/11.67 kb and 1/11.79 kb, respectively, while in *Kernera saxatilis* SSRs were more distanced with 1/35.28 kb (Table 3). Even if the total number of SSRs and their density varied greatly, we detected a consistent pattern in the proportion of each repeat type (i.e., dimers, trimmers, tetramers, pentamers, and hexamers) across species (Figure 2). Our analyses showed that dimmers and trimmers accounted for three quarters of the discovered SSRs (30.66 and 44.43%, respectively) while tetramers and pentamers were scarce (4.35% and 8.09%) and hexamers displayed an intermediate frequency (12.33%). As expected, the number of SSRs with primers (2012) dramatically decreased representing 8.6% of the total. *C. pyrenaica* retained the largest amount of SSRs (160) but, as noted before, this sample was sequenced deeper. Regarding the remaining samples, *M. perfoliatum* maintained 135 SSRs while *D. ossetica* had the smallest number, 61 (Table S1). The primer design parameters also impacted the proportions of the different repeat types. Although dimers and trimmers were still the most abundant accounting for 25.94 and 57.36%, respectively, the frequencies of the remaining SSR types changed. Tetramers increased their frequency to 13.19% while pentamers and hexamers decrease their frequency to 2.52 and 2.96% each (Table S1). The latter indicates that the primers search parameters introduced a bias. Hence, we conducted the analyses concerning SSRs evolution across lineages with the whole SSRs data set instead of with the one containing only those SSRs with primers. Interestingly, the average percentage of SSRs for which we successfully designed primers was higher for the samples sequenced with the old HiSeq platform (avg. 14.14 SSRs) than for the ones sequenced with Illumina HiSeq2000 (avg. 7.28 SSRs), even if the starting number of SSRs was higher in the latter.

**Figure 3:**
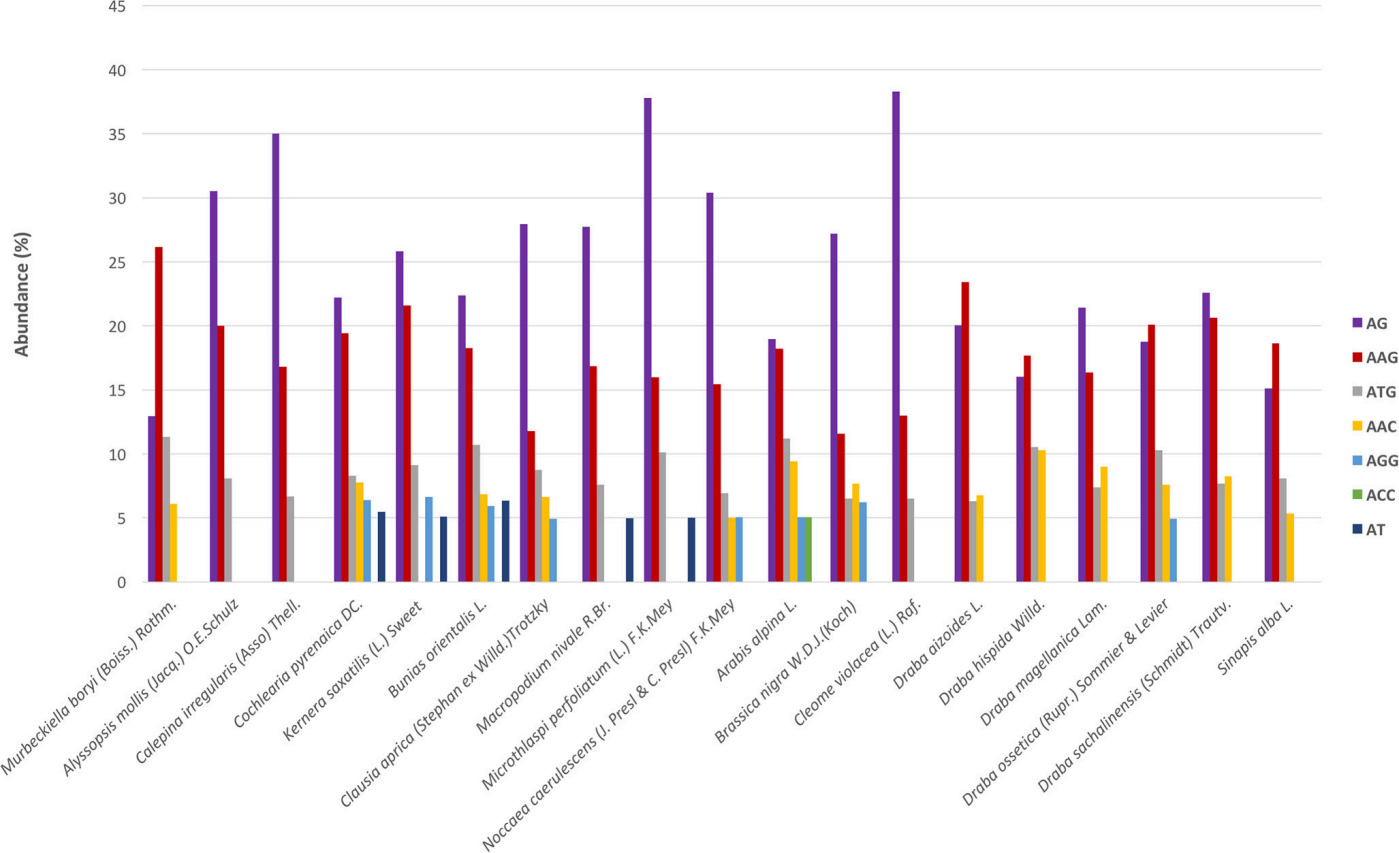
SSR's motifs distribution across species. The x-axis indicates species and the y-axis abundance in percentage. Each motif is denoted by a color as shown in the legend. Bars represents those motifs with ≥5% abundance. These motifs represent, depending on the species, between 55 and 75% of the total motifs' frequency.

Regarding the distribution of motifs, in our analyses we only considered those which accounted for at least 5% of the total. We clustered the motifs based on their sequences (Lopez et al., 2015) and the most abundant groups were AG, AAG, and ATG (Figure 3). These three were present in all species and displayed high frequencies. Moreover, we also detected motifs from the AAC, AGG, ACC, and AT groups but in a subset of species and with lower frequency (Figure 3). Overall, AG was the most abundant dimer while AAG was the commonest trimer. We also looked for variations across the phylogenetic lineages in terms of frequency of the different repeats' and motifs' types. We found no differences suggesting the absence of evolutionary patterns specific to each lineage but rather pointing to a conserved and more general scheme that encompasses the whole Brassicaceae family.

### Impact assessment of the sequencing platform

Since our data set is very heterogeneous, we tested the effect that the number of reads and genome size might have on the *de novo* assembly, as well as in the amount of mined SSRs and their frequency across the transcriptome. Because of the existing variation in terms of sequencing platform we also considered the effect that this interaction might have had on the aforementioned tests. The sample *C. pyrenaica* was excluded from these analyses because of its greater sequencing depth. We corroborated that the sequencing platform has a significant impact on the initial number of reads but not on the number of assembled contigs. The overall number of detected SSRs was dependent on the starting number of reads and therefore biased by the sequencing platform but not by the number of contigs. Namely, those species sequenced with the old Illumina HiSeq rendered a smaller number of SSRs. Likewise, the density of SSRs along the transcriptomes was significantly biased by the sequencing platform. Eventually, we also considered the role of DNA content measured as the 1C number. In this regard, DNA content showed marginal significance for the initial number of reads but not in any other aspect. These analyses further confirmed that sequencing platform exerts a significant impact on number and density of mined SSRs.

### Transcriptome annotation of *Cochlearia pyrenaica*

The *de novo* assembly of *C. pyrenaica* generated 51,572 transcripts belonging to 37,394 Trinity genes (Table 2). Based on the representation of reads and ortholog completeness, we established that our transcriptome had a high quality (83.91% properly mapped reads and 94.46% retrieved orthologs). After the homology search a large proportion of our transcripts (79.40%) had a significant hit to closely related species such as, *Eutrema salsugineum, Camelina sativa, Brassica napus, A. thaliana, Arabidopsis lyrata, Brassica rapa, Arabis alpine*, and *Capsella rubella*. The remaining 20.60% did not match any known sequence and could be novel transcripts, untranslated regions, non-coding RNA or short sequences not containing a protein domain. The Gene Ontology (GO) categorizations we recovered from the transcripts with a significant hit in the homology search were distributed in the three main GO terms in the following order: biological process (68,585), molecular function (46,819), and cellular component (43,132). In the biological process category, most of the transcripts were assigned to metabolic and cellular process. In the cellular function category, the most abundant classes were cell and cell part, while binding and catalytic activity were the classes with the highest number of assigned transcripts in the molecular function category. We successfully annotated 62% of the *de novo* assembled sequences. Of the unannotated portion (~38%), 7% had GO mappings, 10% had Blast hits and 21% had neither GO mapping nor Blasts hits. Within that 21%, we assigned 11 sequences with a significant hit for non-coding RNA in the Rfam database. Five sequences belonged to snoRNAs, two were labeled as plant_SRP and the remaining three were tRNA, SSU-rRNA and miRNA. Eventually, to identify active metabolic pathways, we assigned the transcripts with retrieved GO terms to KEGG pathways and enzyme commission (EC) numbers. We retrieved a total of 6 EC classes with transferases (3255) and hydrolases (2191) as the most abundant. We included the EC numbers in KEEG pathways identifying biosynthesis of antibiotics (697 sequences with 158 enzymes) and purine metabolism (512 sequences wit 47 enzymes) as the most represented pathways. Other well-characterized pathways were starch and sucrose metabolism (298 sequences with 31 enzymes), pyrimidine metabolism (236 sequences with 27) and amino sugar and nucleotide sugar metabolism (221 sequences with 35 enzymes). Finally, we added the ORF prediction to the annotation results with 24,487 complete ORFs, 3393 partial ORFs and 3911 internal ORFs. Overall, we provide a high quality and well annotated transcriptome which represents a valuable tool for future studies aiming to disentangle the evolutionary history of the plant genus *Cochlearia*.

### SSRs transferability and polymorphism in the genus *Cochlearia*

We identified a total of 2738 SSRs in the species *C. pyrenaica* (Table 3) and from those 160 fulfilled our primer search criteria. Trimers were the most common type of SSR followed by dimers, both before and after primer search (Table S1). Motifs from the AG group were the most abundant (22.21%) followed by the group AAG (19.44%; Figure 3). We crossed the 160 SSRs with primers with the transcriptome's annotation showing that 74 were located within ORF and 86 outside (Table 4). When analyzed in detail, the SSRs were not equally distributed across the transcriptome. We found that trimers clearly dominated within ORFs (>85%) while dimers and tetramers contributed the most (>82%) outside ORFs.

**Table 4:** ORF location and type distribution of SSRs used for the *in silico* polymorphism test in *Cochlearia* species.

	**Dimers**	**Trimers**	**Tetramers**	**Pentamers**	**Hexamers**	**Total**
In-ORF	5	63	4	0	2	74
Out-ORF	34	18	28	4	2	86
Total	39	81	32	4	4	160

*In-ORF indicates SSR located within coding region while out-ORF denotes SSR outside coding region*.

For the *in silico* polymorphism evaluation, we randomly selected 16 SSRs which were we tested in 13 individuals from seven species of the genus *Cochlearia* (Table 2). Our species data set included six individuals from *C. pyrenaica*, two individuals of *C. groenlandica* and one individual of each of the remaining five species. Regarding the SSRs, eight were located within ORFs (seven trimers and one hexamer) and eight outside ORFs (three dimers, two dimers and two tetramers; Table 5). We mapped genomic reads of the different *Cochlearia* species to the *C. pyrenaica* transcriptome and selected those mapped regions with the transcripts containing SSRs. In order to find the expected PCR product, we performed a search of the SSR for both primers, forward and reverse. By identifying the whole PCR product, we could also detect presence of introns or other elements that might modify the expected fragment length. In this regard, none of the 16 selected SSRs deviated from the expected length. Moreover, the primers' sequences were conserved in all species indicating that these SSRs are fully transferable across all tested species in the genus *Cochlearia*.

**Table 5:** SSRs' information for the *in silico* polymorphism study in *Cochlearia* sp.

**ID**	**ORF**	**P_o_**	**P_f_**	**SSR**	**Annotation**
c12574_g3_i1	in	379	399	CAA	Uncharacterized protein.
c13603_g4_i1	in	417	437	GGT	NADH-ubiquinone oxidoreductase 20.9 kDa subunit-like.
c13427_g2_i1	in	482	502	TTC	Glucose-6-phosphate isomerase 1, chloroplastic.
c13710_g1_i1	in	227	247	TCT	Protein with domain DUF314 -unknown function.
c13881_g1_i2	in	1844	1864	CCA	Phosphatidylinositol-4-phosphate 5-kinase 1.
c147_g2_i1	in	159	179	GTG	Transcription factor TCP15-like.
c15301_g1_i1	in	330	371	CAAAAC	Uncharacterized protein.
c7760_g1_i1	in	276	296	GGT	Uncharacterized protein with domain DUF616.
c13912_g1_i1	out	278	298	TCA	No hit in homology search.
c13000_g1_i1	out	1112	1132	GTA	No hit in homology search.
c14456_g1_i1	out	112	131	AT	No hit in homology search.
c16762_g1_i1	out	248	267	AC	No hit in homology search.
c25249_g1_i1	out	2453	2472	AG	No hit in homology search.
c28231_g1_i1	out	135	155	ACG	No hit in homology search.
c10839_g2_i2	out	762	781	ATGG	No hit in homology search.
c13423_g2_i1	out	193	212	TGCT	No hit in homology search.

*ID, as contig identifier; ORF, “in” designates SSR located in ORF while “out” points to SSR outside ORF; P_0_, denotes starting position of the PCR product in contig while Pf last position; SSR, indicates length and sequence of the SSR and annotation, explains the description retrieved from the annotation process*.

**Figure 4:**
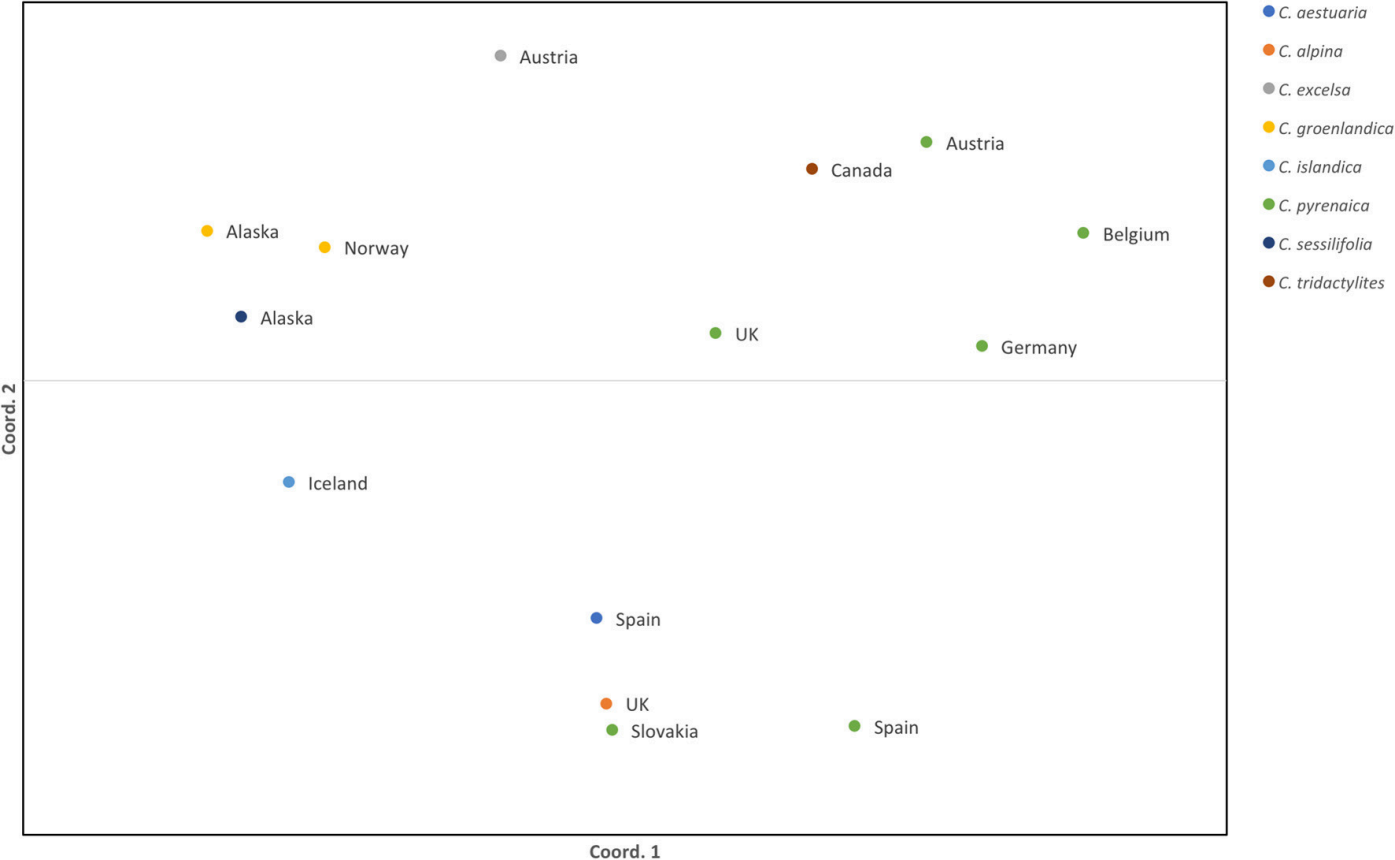
Standardized distance PCoA for Cochlearia samples. Each color represents a species from the genus *Cochlearia* and the country of origin is displayed next to the sample symbol. Both coordinates explain 51.24% of the variability (coord.1 accounted for 32.27% and coord.2 18.97%).

Once the PCR product was identified we scored the SSRs. Because of possible PCR inaccuracies, we only considered an allele as “true” if it was present in at least two reads. One sample, Cpyr_0699 had no reads mapped to two SSR-containing contigs and therefore those were coded as missing alleles (Table 6). As expected for diploid species, the maximum number of alleles we found per SSR was two (Table 6). Despite the reduced number of individuals, and with the only purpose to be used as guidance, we computed several diversity measurements. Overall, mean expected heterozygosity (H_e_) was 0.210 (SE = 0.023) and the mean percentage of polymorphic loci was 40.63% (SE = 9.67%). In terms of species' diversity our only sample of *C. sessilifolia* was monomorphic for all scored SSRs and thus, the polymorphism at species level was zero. The highest percentage of polymorphic loci was 87.50% in *C. pyrenaica* followed by *C. groenlandica* (56.25%). These two species were the ones with more than one individual per species scored. In the case of *C. pyrenaica* we analyzed six individuals and its genetic diversity can be considered as realistic measurement (H_e_ = 0.512, SE = 0.068). For the remaining one-individual-per-species samples polymorphism ranged from 25.00 to 56.25%. Because of the reduced *n* per species these measurements cannot be considered an accurate representation of the species' diversity. SSRs in coding regions are expected to be more conserved thus, we tested if they displayed lower levels of polymorphism than those in non-coding regions. We re-run the diversity analysis for the SSRs within ORFs (coding) vs. those outside the ORFs (non-coding). In the first case of coding regions, the H_e_ recorded was 0.230 (SE = 0.033) and mean polymorphism of 45.31% (SE = 10.28%). Interestingly, in non-coding SSRs the total mean H_e_ was 0.189 (SE = 0.033) and mean polymorphism of 35.94% (SE = 10.96%). Finally, the first two axes of the PCoA calculated for the individuals based on their genetic distance explained 51.24% (coord.1 accounted for 32.27% and coord.2 18.97%). The six individuals of *C. pyrenaica* formed a dispersed cluster while the two individuals of *C. groenlandica* grouped tightly together (Figure 4). The PCoA also revealed a geographical sub-grouping where most individuals from Artic and Alpine areas clustered together (Alaska, Iceland, Norway, and alpine Austria). Our transferability and polymorphism analyses showed that this is a suitable approach for developing large sets of SSRs for population studies within and across related species.

**Table 6:** SSR polymorphism in species from the genus *Cochlearia*. Alleles scored in each sample per SSR (indicated by length in bp).

	**Caes_0741**	**Calp_0759**	**Cexc_1253**	**Cgro_0474**	**Cgro_1038**	**Cisla_1233**	**Cpyr_0260**	**Cpyr_0310**	**Cpyr_0456**	**Cpyr_0560**	**Cpyr_0699**	**Cpyr_1211**	**Cses_1285**	**Ctri_1287**
c12574_g3_i1	18–21	21	21	18	15–18	15–18	15	21	18	18–21	18	21–24	18	18
c13603_g4_i1	9–12	12–24	12–15	21	12	24	24	12	12	15	18–24	12	21	21
c13427_g2_i1	18–21	21	21–36	21–27	21	21–27	27–33	21–32	21	21	12	21	21	27
c13710_g1_i1	18	18	18	18	18	18	24	18	18	21	15–18	12	18	18
c13881_g1_i2	18–21	15–18	18–21	18	21	18	21	21	21	21	9–18	21	18	9–18
c147_g2_i1	21	12	12	21	12	21	15	12	12	12	12	12	21	12–24
c15301_g1_i1	18	18	18	18	18	18	18	18	18	18	18	18	18	18
c7760_g1_i1	18–21	21	27–30	18–21	15	21	27	18–27	18	21	18–21	18	21	15–27
c13912_g1_i1	12–18	18	18	18	18	18	18	18	18	18	18	18	21	15–18
c13000_g1_i1	15	15–21	15–18	15	15	15	12	18	15	12	18–21	12–15	15	18–21
c14456_g1_i1	10	16	14	18	20	10	18	14	16	16	10–16	16	16	14–16
c16762_g1_i1	20–22	18	24	18–26	18	18	14	20	20–22	18	12–26	14	20	18–24
c25249_g1_i1	16–18	18	16	16	16	16	22	24	10	14	NA	18	16	14
c28231_g1_i1	15	15	18	9	9	15	12–18	12–24	15	18	NA	15	9	9–15
c10839_g2_i2	16	20–24	24	20–24	24	24	16	16	20	16	24–28	16–28	24	12–20
c13423_g2_i1	20	20	20	20	20	12–20	16–20	20	20	20	20	20	20	16

## Discussion

The Brassicaceae is a captivating model for evolutionary studies. This family is one of the biggest in the plant kingdom with taxa distributed worldwide including model species and economically relevant crops. Besides, it shows a complex history with high rates of speciation and recurrent polyploid events distributed across its phylogeny at both ancient and more recent time scales. Nevertheless, there is still more to learn from this family. In the present study, we developed a large amount of SSRs with their respective primers (2012) in 19 Brassicaceae species from their *de novo* assembled transcriptomes. Besides, we used eight species from the genus *Cochlearia* to test their transferability and polymorphism *in silico*. In this genus, we went one step further and also annotated its transcriptome. Overall, we provide a new set of tools for evolutionary studies in the family Brassicaceae and we discuss its implications.

### Impact assessment of the sequencing platform

During the design of an experiment, choices like the sampling strategy or the selection of a particular molecular tool can deeply impact the outcome. In studies using NGS technologies, the chosen sequencing platform could be one of these determinant factors. However, there is no clear consensus regarding how strongly its influence can be. In some cases, the sequencing platform resulted in little or no after-affect (Solonenko et al., 2013; Tremblay et al., 2015). In contrast, other studies demonstrated a significant bias in relevant aspects like the G = C content of the data among others (Benjamini and Speed, 2012; Salipante et al., 2014). Our results strongly support that the sequencing platform exerts a strong bias on the results of studies using NGS data for SSR mining in aspects like overall number of mined SSRs or SSR density suggesting caution when data from different sequencing platforms are combined.

### Compositional analysis of SSR mining in the brassicaceae family

The frequency and distribution of SSRs from the transcribed regions of the genome can vary greatly among studies as their discovery depends on aspects such as, mining criteria, sequencing platform, or the completeness of the inputted transcriptome (Aggarwal et al., 2006; Blair and Hurtado, 2013). In an effort to increase polymorphism and avoid fluctuations in the length of the PCR product not associated with the SSR *per se*, we choose conservative criteria and only perfect SSRs with at least 20 bp with no other SSRs in the flanking or primer regions were considered (Blair and Hurtado, 2013). Hence, if we had used more relaxed parameters for the searching, the amount of obtained SSRs could have been larger. Overall, we found 23,425 SSRs in 19 Brassicaceae species. Those 19 species covered all lineages of the Brassicaceae phylogeny projecting a comprehensive picture of the family. The overall number of SSRs and their density along the *de novo* assembled transcriptomes varied largely across species. These changes have been attributed in some cases to genome size (Qiu et al., 2010). However, in our case these changes seem to be caused by platform sequencing rather than genome size. In fact, when we considered only the data set generated by Illumina HiSeq2000 and looked for correlations between the 1C number with the number of SSRs or the SSRs density per species, no clear pattern emerged. Despite the fact that the sequencing platform influenced the overall amount of mined SRRs and their density, the general distribution of the different SSR types and motives remained stable. Trimers, followed by dimers, were the most abundant motives accounting for ~75% while tetra-, penta- and hexa-nucleotides had lower frequencies in all studied species. This pattern has been previously reported for numerous angiosperm taxa (Kantety et al., 2002; Morgante et al., 2002; Victoria et al., 2011). Likewise, the most common dimer motif was AG/CT while the rarest was the CG/GC as in similar studies with *Arabidopsis*, rice and several other angiosperms (Kantety et al., 2002; Morgante et al., 2002; Victoria et al., 2011; Lopez et al., 2015). The high amount of GA/CT motifs is likely to be related to high levels of the translated amino acid products of these motifs (Kantety et al., 2002; Qiu et al., 2010). Among the trimers, our results also fitted the expectations for angiosperms with AAG/CTT as the most abundant group followed by the ATG/CAT group and the rarest was AAT/TTA (Morgante et al., 2002; Victoria et al., 2011). Few other trinucleotide motives had a frequency of at least 5% but none of them was consistently found across all studied species as the two previous ones were. Finally, besides the motives found in each and all studied species, no motif was represented in a particular phylogenetic lineage.

Microsatellite distribution is known to be a function of the dynamics and history of genome evolution (Morgante et al., 2002). The dominance of trinucleotides responds to the coding nature of the transcriptome as selection and evolution benefit the presence of repeat types which maintain the coding frame (Wang et al., 1994; Gao et al., 2003). Yet, a large number of dinucleotides was also found suggesting that there is a considerable amount of non-coding regions like introns and UTR which might benefit from polymorphism affecting the regulation of transcription expression levels (Gingeras, 2007). Overall, this congruency with previous studies in angiosperms in the frequency pattern of the various SSR repeat types and motives points to the conserved nature of SSR evolution despite the complex and rich evolutionary history of the Brassicaceae family.

The final amount of available SSRs with their respective primers was significantly smaller compared with the total number of mined SSRs as expected from our strict mining criteria. Our data set comprehends 2012 SSRs with their respective primers ranging from 61 to 160 per species. After primer search the general SSR frequency pattern stayed fairly stable with mean percentage of trimers of 59.25 followed by dimers with 25.10% and the remaining repeat types displaying lower frequencies. However, the not-so-common repeat types experienced some changes. In this regard, tetramers went from a mean 5.11 to 12.61% while penta- and hexamers changed from 8.76 to 2.11% and 13.17 to 2.10%, respectively. Primer search, as mining criteria, have an enormous impact on the final size of the data set. Here we opted for a more conservative approach to ensure the stability, polymorphism and transferability of our SSRs instead of aiming for a larger number of mined SSRs which might lead to lower accuracy of the PCR products derived from our SSRs.

### Transcriptome annotation of *Cochlearia pyrenaica*

The genus *Cochlearia* is a non-model system in the Brassicaceae with no prior genome information. This lack of genomic resources hinders the study of its evolutionary history. Thus, besides the SSR discovery, we annotated the *de novo* assembled transcriptome of *C. pyrenaica* in an effort to provide more comprehensive resources for future studies. Circa 80% of the transcripts had a positive hit on the protein databases indicating that most of our transcripts coded proteins. The latter was probably facilitated by the annotated genomes from close relatives of the Brassicaceae family such as, *A. thaliana* or *E. salsugineum*. For those transcripts without any hit during the annotation process we ran a Rfam search. Only 11 transcripts had a match. Therefore, most of our unidentified transcripts are probably lacking conserved functions or might belong to uncharacterized genes. Overall, based on our quality measurements and the large proportion of annotated transcripts, we are confident of the accuracy and completeness of our *de novo* assembled and annotated *C. pyrenaica* transcriptome.

The GO terms association provides an important resource for the identification of gene roles (Ashburner et al., 2000). Overall, we assigned 159,836 GO terms and classified them into the three main categories (biological process, cellular component and molecular function). Within the biological process, several GO terms (e.g., cold adaptation and salt tolerance) are of special relevance for the evolutionary history of *Cochlearia* and open a new horizon for future studies. This genus is known to inhabit a wide range of environments (salt marshes, high alpine environments, and artic regions among others) but the mechanisms behind these adaptations which might have mediated the expansion of the genus are unknown (Koch et al., 1998, 1999; Koch, 2012). Besides, several pathways we detected that in the *C. pyrenaica* transcriptome are putatively associated with adaptation to these environments. Namely, starch and sucrose metabolism are known to play a role during cold acclimation (Beck et al., 2007; Peng et al., 2015). Likewise, the identification of enzyme codes in the present study is likely to be helpful for understanding the metabolic activities of the contrasting species in this interesting and emblematic genus.

### SSRs transferability and polymorphism in the genus *Cochlearia*

We selected 14 individuals of eight diploid *Cochlearia* species to conduct a performance test addressing polymorphism and transferability of our SSRs. These species are very interesting because of their complex polyploid and reticulate evolutionary history which spans a few 100, 000 years only (Koch et al., 1998, 1999; Koch, 2002, 2012). Because of their association with conserved parts of the genome, SSRs derived from the transcribed region are often considered to display low levels of polymorphism (Ellis and Burke, 2007). However, numerous studies contrasting SSR from the anonymous and transcribed regions of the genome showed that this premise does not hold true as they show comparable levels of polymorphism (Tiffin and Hahn, 2002; Woodhead et al., 2005). Our within-species measurements cannot be considered an accurate reference as we only had more than one individual per species in *C. groenlandica* (*n* = 2) and *C. pyrenaica* (*n* = 6). Still, our data set involved a comprehensive group of the genus representatives making it suitable to test transferability and polymorphism across species. We considered a SSR transferable across species when both primers were found in the genomic reads. Based on this criterion all 16 SSRs were 100% transferable across species. Even if our search parameters allowed for two bases difference between the primer and targeted sequence, all primers had an identical match suggesting full transferability across species. We found no deviation from the expected inferred PCR product indicating absence of non-transcribed introns (Lopez et al., 2015). However, deviation cannot be completely disregarded for all mined SSRs as we tested only a small fraction.

The expected heterozygosity detected for *C. pyrenaica* falls within the expected for short-lived perennial plants (Nybom, 2004) supporting the adequacy of these type of markers in studies addressing genetic variation within species. When we considered all species, the expected inter-species heterozygosity decreased (H_e_ = 0.230, SE = 0.033). The latter could be explained by presence of several individuals with reduced polymorphism. Life history traits are known to have a strong impact on the species' genetic variation and population structure (Nybom, 2004) and our lower inter-species measurement can also be a consequence of contrasting features of the species included in our data set. The computation of F_ST_ was impeded by the low number of individuals per species. Thus, we performed a PCoA as an approximation of genetic structure. In the PCoA individuals from the same species appeared tightly clustered for *C. groenlandica* while for *C. pyrenaica* the cluster was more dispersed. Interestingly, the two individuals of arctic *C. groenlandica* were grouped with arctic C. *sessilifolia* from Alaska, arctic *C. islandica* from Iceland and alpine *C. excelsa* from Austria forming an arctic/alpine environmental deme. We also observed a geographical clustering in *C. pyrenaica* where individuals from central Europe and UK are closer together compared with individuals from Spain. Surprisingly the individual from Slovakia was closer to the Spanish accession that to the Central European ones. Another striking detail was the position of the Canadian sample which, due to environmental conditions, we expected to be grouped with the other Arctic/Alpine samples. Instead, it was positioned in the Central European deme. These two anomalies could be attributed to the limited number of samples and SSRs used for this test. Because of the latter we cannot make general assumptions but, based on our PCoA, it seems reasonable to say that this approach for SSRs development can also be regarded as an alternative to classical SSRs when inferring species' genetic structure.

Finally, the annotated transcriptome of *C. pyrenaica* allowed us to identify the location of the SSRs in coding and non-coding regions. Based on the premise that coding parts are conserved, we expected SSRs from these to be less polymorphic. Interestingly, we found that SSRs located within ORF displayed higher levels of diversity than their non-coding counterparts. Our data set comprehends multiple species distributed worldwide which inhabit contrasting environments and are likely to be adapted to their local environments. Thus, the higher diversity observed in the SSRs from coding regions may be attributed to adaptation and selection processes. Overall, we have established a significant genomic data resource for the genus *Cochlearia* providing a comprehensive annotated transcriptome and a large set of polymorphic molecular markers suitable for genetic studies which are transferable between species.

## Conclusions

Our main goal in this study was to identify genic-markers that can be immediately available in evolutionary studies across the Brassicaceae family. Among various molecular markers we choose SSRs because they are extensively and successfully used for genetics and plant breeding applications (Hiremath et al., 2012). Overall, we identified 2012 SSR markers with their respective primers for 19 Brassicaceae species which are publicly available in the BrassiBase portal (Kiefer et al., 2014). As shown in our transferability test with the genus *Cochlearia* these transcriptome-based markers are fully transferable within the genus. Even more, several studies have found that transferability can go beyond the within-genus level (Varshney et al., 2005b) increasing exponentially the number of targeted species. Our polymorphism test supports previous studies indicating that these markers have similar variation levels as classical SSRs (Tiffin and Hahn, 2002; Woodhead et al., 2005). Also, we found that the SSRs within coding regions harbored larger variability than the non-coding ones suggesting that these markers might be suitable to detect functional adaptive variation across populations and/or species. Additionally, we delivered a high quality annotated transcriptome for *C. pyrenaica* which facilitates future evolutionary studies in this fascinating genus. Our results showed that sequencing platform has a significant impact on the SSRs discovery outcome and we recommend that when combining different data sets this bias should not be taken lightly. Finally, SSRs' evolutionary patterns across Brassicaceae lineages are highly conserved despite its complex evolutionary history and all our findings were in agreement with previous studies conducted in other Angiosperms.

## Data availability

The assembled transcripts used for the SSR mining and further analysis are available in Dryad (https://doi.org/10.5061/dryad.35k67). Likewise, the file containing the annotated transcriptome can be found at Dryad (https://doi.org/10.5061/dryad.35k67). Finally, the SSR with their respective primer and all accompanying information can be found at the Brassibase (XXX) and Dryad (https://doi.org/10.5061/dryad.35k67).

## Author contributions

LL and MK designed the study and set up the analysis. LL processed and analyzed the data. EW, PE, and JP made relevant contributions to the data analysis process. LL drafted the manuscript and MK, EW, PE, and JP critically revised the manuscript. All authors read and approved the final version of the manuscript.

### Conflict of interest statement

The authors declare that the research was conducted in the absence of any commercial or financial relationships that could be construed as a potential conflict of interest.
