# Supplementary material for: Molecular Resources from Transcriptomes in the Brassicaceae Family
Source: Front Plant Sci. 2017 Aug 29;8:1488. doi: 10.3389/fpls.2017.01488 (PMC5581910; doi:10.3389/fpls.2017.01488)
Supplement: Supplementary file 1 [file Table1.DOCX]

**Supplementary Material**

| **Species** | **Dimers** | | **Trimers** | | **Tetramers** | | **Pentamers** | | **Hexamers** | | **Total** | |
| --- | --- | --- | --- | --- | --- | --- | --- | --- | --- | --- | --- | --- |
|  | **T** | **w/P** | **T** | **w/P** | **T** | **w/P** | **T** | **w/P** | **T** | **w/P** | **T** | **w/P** |
| *Murbeckiella boryi* (Boiss.) Rothm. | 189 | 30 | 568 | 83 | 22 | 9 | 91 | 1 | 135 | 5 | 1005 | 128 |
| *Alyssopsis mollis* (Jacq.) O.E.Schulz | 659 | 34 | 735 | 60 | 87 | 14 | 140 | 0 | 187 | 3 | 1808 | 111 |
| *Calepina irregularis* (Asso) Thell. | 615 | 30 | 565 | 49 | 51 | 12 | 124 | 6 | 161 | 3 | 1516 | 100 |
| *Cochlearia pyrenaica* DC. | 831 | 39 | 1415 | 81 | 192 | 32 | 217 | 4 | 128 | 4 | 2783 | 160 |
| *Kernera saxatilis* (L.) Sweet | 430 | 29 | 595 | 71 | 41 | 9 | 74 | 3 | 176 | 3 | 1316 | 115 |
| *Bunias orientalis* L. | 455 | 25 | 728 | 64 | 60 | 14 | 120 | 3 | 170 | 6 | 1533 | 112 |
| *Clausia aprica* (Stephan ex Willd.)Trotzky | 481 | 26 | 538 | 50 | 44 | 13 | 132 | 4 | 223 | 3 | 1418 | 96 |
| *Macropodium nivale* R.Br. | 811 | 33 | 915 | 56 | 134 | 21 | 263 | 7 | 401 | 11 | 2524 | 128 |
| *Microthlaspi perfoliatum* (L.) F.K.Mey | 594 | 34 | 523 | 80 | 30 | 17 | 44 | 1 | 103 | 3 | 1294 | 135 |
| *Noccaea caerulescens* (J. Presl & C. Presl) F.K.Mey | 818 | 47 | 787 | 59 | 54 | 18 | 150 | 3 | 286 | 3 | 2095 | 130 |
| *Arabis alpina* L. | 168 | 27 | 415 | 71 | 27 | 8 | 54 | 3 | 131 | 1 | 795 | 110 |
| *Brassica nigra* W.D.J.(Koch) | 225 | 33 | 289 | 72 | 41 | 20 | 60 | 3 | 76 | 2 | 691 | 130 |
| *Cleome violacea* (L.) Raf. | 675 | 18 | 485 | 58 | 66 | 15 | 91 | 2 | 131 | 4 | 1448 | 97 |
| *Draba aizoides* L. | 96 | 24 | 198 | 50 | 16 | 5 | 33 | 0 | 72 | 2 | 415 | 81 |
| *Draba hispida* Willd. | 82 | 21 | 219 | 43 | 19 | 5 | 43 | 2 | 55 | 1 | 418 | 72 |
| *Draba magellanica* Lam. | 115 | 15 | 194 | 37 | 26 | 10 | 33 | 1 | 66 | 1 | 434 | 64 |
| *Draba ossetica* (Rupr.) Sommier & Levier | 105 | 17 | 213 | 37 | 18 | 4 | 47 | 1 | 65 | 2 | 448 | 61 |
| *Draba sachalinensis* (Schmidt) Trautv. | 202 | 24 | 333 | 38 | 39 | 13 | 55 | 3 | 88 | 2 | 717 | 80 |
| *Sinapis alba* L. | 168 | 15 | 364 | 61 | 63 | 23 | 100 | 2 | 73 | 1 | 768 | 102 |

**Table S1**: *SSR distribution across species*. Number of dimers, trimmers, tetramers, pentamers, hexamers, as well as, total number of SSR discovered in the *de novo* assembled transcripts. Each category is divided in T, indicating total number of SSR and w/P referring to those SSR where we successfully designed primers.
